# Trapping and assembling of particles and live cells on large-scale random gold nano-island substrates

**DOI:** 10.1038/srep09978

**Published:** 2015-04-30

**Authors:** Zhiwen Kang, Jiajie Chen, Shu-Yuen Wu, Kun Chen, Siu-Kai Kong, Ken-Tye Yong, Ho-Pui Ho

**Affiliations:** 1 Centre for Advanced Research in Photonics, Department of Electronic Engineering, The Chinese University of Hong Kong, Shatin, N.T., Hong Kong SAR, China; 2 Program of Biochemistry, School of Life Sciences, The Chinese University of Hong Kong, Shatin, N.T., Hong Kong SAR, China; 3 School of Electrical and Electronic Engineering, Nanyang Technological University, Singapore 639798, Singapore

## Abstract

We experimentally demonstrated the use of random plasmonic nano-islands for optical trapping and assembling of particles and live cells into highly organized pattern with low power density. The observed trapping effect is attributed to the net contribution due to near-field optical trapping force and long-range thermophoretic force, which overcomes the axial convective drag force, while the lateral convection pushes the target objects into the trapping zone. Our work provides a simple platform for on-chip optical manipulation of nano- and micro-sized objects, and may find applications in physical and life sciences.

Plasmonic tweezers are devices that harness enhanced optical gradient force exerted on targets (e.g. micro- or nano-objects) when they interact with the tightly localized near-field of surface plasmons^1^. Due to their near-field characteristics, the spatial-resolution of device is not confined by diffraction-limit. Moreover, field amplification associated with surface plasmons also significantly reduces the trapping power threshold by three to four orders of magnitude^2^ upon comparing to conventional optical tweezers^3^. Optical manipulation with plasmonic effects offers a broad range of attractive applications in biochemistry^4^, sensing^5^, and on-chip microfluidic cell sorting^6^.

By carefully engineering the metallic nanopatterns, one can control the excitation of localized surface plasmons (LSP) and the subsequent trapping potential well for controllable organizing of micro- and nano-objects. So far, different types of objects such as metal nanoparticles^5^, living organism^6^, dielectric particles^7^,^8^,^9^, and DNA^10^ were trapped and organized into the form of single molecule or clusters. Various functional plasmonic patterns including dipole nanoantenna^5^, bowtie arrays^7^, square optical lattice^8^, nanopyramidal-dimer arrays^9^,^10^, disk^6^,^11^, and cavity^12^ have been reported in the literature. All these patterns require high-precision fabrication techniques that are based on electron-beam lithography (EBL)^5^,^6^,^7^,^8^, colloidal lithography^9^,^10^, or focused-ion beam (FIB) milling^12^. However, such fabrication techniques, especially the EBL and FIB, are time-consuming, expensive and produce low yield of products. As such, it is not practical to employ plasmonic tweezers for large-scale applications when only limited plasmonic patterns can be produced per batch of fabrication. Colloidal lithography is an established low-cost, large-scale nanofabrication technique based on nanospheres, but this technique still requires lithographic steps such as spin coating and lift-off processing. Although plasmonic trapping in continuous metal films through the excitation of surface plasmon polaritons (SPPs) can be achieved^13^, the setup requires specific optical geometry alignment (viz., the Kretschmann configuration) and the incident angle for activating the trapping mode has a narrow window, thus making this approach not compatible with common optical microscopy instruments.

Speckle, also known as random light field, is a common interference pattern resulted from multiple scattering of light in optically complex media such as on rough surfaces^14^. Although random and sometimes regarded as nuisance, the potential wells resulted by speckle can be used to conduct controllable micromanipulation of objects in various mediums^15^. Recent developed speckle optical tweezers can achieve trapping, guiding, and sieving of objects theoretically by using static and time-varying speckle light fields^16^, which therefore exhibits promising perspective for the real-life applications in biophotonics and nanomedicine.

Inspired by this concept, we propose the use of plasmonic random systems to realize trapping, with the stochastic localized field of surface plasmons analogical to the near-field speckle. Typically, such random structures sample is taking the form of gold nano-island substrate (AuNIS), which can be easily prepared by using metal deposition followed by thermal annealing. Our experiments have revealed that trapping, manipulating, and stacking of polystyrene spheres (PS, nm to μm in size) are achievable with very low power density. We also have successfully applied this approach for trapping and organizing *Escherichia coli* cells. In our system, local heating induced thermal forces (long-range interaction) including convective drag force and thermophoretic force always go alongside the optical trapping force (short-range interaction). While lateral convection supports the trap by transporting far-field targets into the trapping zone, optical trapping force is superimposed with a thermophoretic force attributed to pushing the PS particles from cold to hot regions. If the over effect of these two components overcomes the axial convective drag force, a net force towards the surface is established, thus making the trap stable for a long period of time. Our results show that the random gold nano-island is a very effective substrate for trapping and manipulation applications. One can simply direct a laser beam at this substrate to activate the trapping mode. This is a major advancement in plasmonic tweezers as conventional ones are solely based on well-defined metallic nanostructures.

## Results

**Figure 1:**
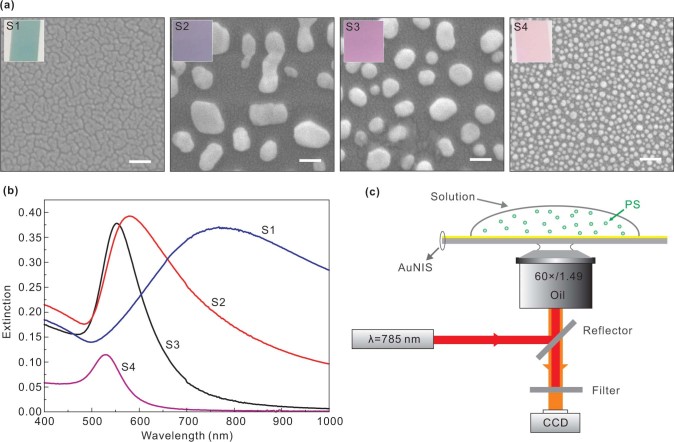
(a) Scanning electron microscope (SEM) images of S1-S4. White bar: 100 nm. The insets show the characteristic colors of the samples under white light illumination. (b) Extinction spectra of S1-S4. (c) Schematic of optical setup for the experiments.

Figure 1(a) show the scanning electron microscopy (SEM) images of four different AuNIS samples S1–S4. These samples clearly contain disordered cracks for S1 and random distributed nanoparticles for S2–S4. The cracks (or nanogaps) in S1 have a feature size of around 10 nm as obtained through analyzing the SEM images. The size distributions of the nanoparticles are 80–300 nm for S2, 30–140 nm for S3, and 15–35 nm for S4, respectively. Because of the morphological variations of the nanostructures which are crucial to the resonances, the extinction spectra of S1–S4 are totally different [Fig. 1(b)], and leading to the unique distinctive colors of the samples [insets of Fig. 1(a)]. The inhomogeneous features such as island shapes, aspect ratios and circularity, and the particle size distribution, significantly affect the collective optical responses of the samples^17^. The resonant peaks for S1–S4 are determined to be 768, 578, 552, and 528 nm, respectively. The bandwidth of S3 and S4 are relatively narrow, suggesting that the gold islands in these two samples exhibit a lower aspect ratio and thus more isotropic shapes^17^. Figure 1(c) schematically shows the optical setup, where the trapping laser power on the nano-islands is adjustable between five levels, with P1–P5: 1.0, 3.7, 9.2, 14.5, and 41.6 mW, respectively.

**Figure 2:**
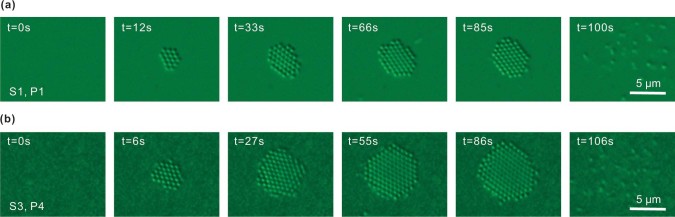
Successive image frames obtained during the trapping of PS particles by S1 at P1 (a), and by S3 at P4 (b), respectively. The laser beam was switched on at t = 0 s, and switched off immediately before the last image. Also see Supplementary Movie 1 as an example.

Our experiments show that S1, S2, and S3 exhibit similar optical trapping behavior. Representative optical trapping images [Figs. 2(a) and 2(b)] show that it takes several seconds for the PS to migrate and confine within the trapping region which is induced by the laser illumination. It was found that the trapped PS organized themselves into a tightly packed hexagonal assembly. As soon as the laser spot is removed, the trapped PSs disperse away from the trapping zone within seconds (see Supplementary Movie 1 as an example). In addition, we found that S3 is able to trap more PS particles at a higher laser power, even though it has comparatively weaker resonant strength at λ = 785 nm when compared to S1 [Fig. 1(b)]. We will discuss this observation later. It should be pointed out that 14.5 mW [P4, Fig. 2(b)] is regarded as high power in the present case. However, because of the large area of the laser spot (diameter of 12 μm), the laser power density within the trapping region is as low as 128.2 *μW/μm*^2^. Trapping did not occur in S4 since it has weak response at λ = 785 nm.

**Figure 3:**
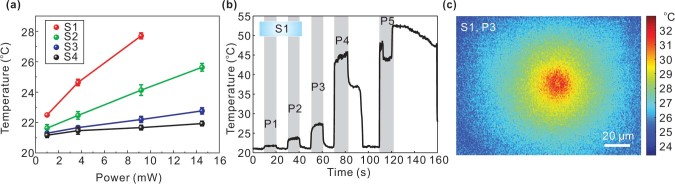
(a) Temperature variations for the AuNIS (S1-S4) as a function of laser power. The error bars denote the standard deviations of five repeated measurements. (b) Temporal responses of temperature as a function of time when the laser power was changed on S1. The anomalous fluctuation of temperature at P4 and P5 on S1 is due to the steam bubble generation. (c) Temperature distribution profiles over the surfaces of S1 at P3. The room temperature was 21^o^C.

It is worth noting that the trapping laser beam also induces thermal effect because of the local heating generated through plasmonic absorption, especially when the structure is in resonance state. Figure 3 shows the detailed surface temperature variations of samples S1–S4 under laser illumination. The local temperature is always highest on the surface of nanostructure and quickly decayed away from surface^18^. In the trapping experiments, the observed convection current increases the trapping zone considerably by allowing one to draw the PS from a greater distance (>30 μm) towards the trapping region. Generally, local heating results in a large temperature gradient that leads to convection and thermophoresis^18^. The thermal convection has a typical toroidal shape, with the fluidic current flowing radially in and out of the hot region, with the in-flow parallel to the AuNIS surface and out-flow vertical to hot zone^19^. On the other hand, the thermophoresis is normally considered to push the particles away from hot zone^9^,^20^,^21^,^22^. However, the magnitude and direction of thermophoresis is very sensitive to the local environment^20^,^21^,^22^,^23^, which implies that the particles might be pushed from cold to hot region under some conditions. In other words, to stabilize and maintain the trapping zone as shown in Fig. 2, a net force towards the surface of AuNIS due to competition between optical and thermal forces, which will be discussed in detail in the Discussion section, has to be present. Indeed, thermal effect assisted optical trapping have been observed recently in plasmonic bowtie nanoantenna arrays^7^,^19^, square optical lattice^8^, and nanopyramidal-dimer arrays^9^. On the other hand, optical and thermal effects will increase with laser power. The net force may change its magnitude gradually and even alter its direction at a threshold, thus the trapping effect will slowly diminish as laser power increases. Further increasing the power will lead to bubble generation (see Supplementary Fig. S1). The trapping outcomes of S1–S4 at P1–P5 are summarized in Supplementary Table S1. For a given incident power level, we also see that the area of the trap increases from S1 to S3, while S4 has no trapping effect at all.

**Figure 4:**
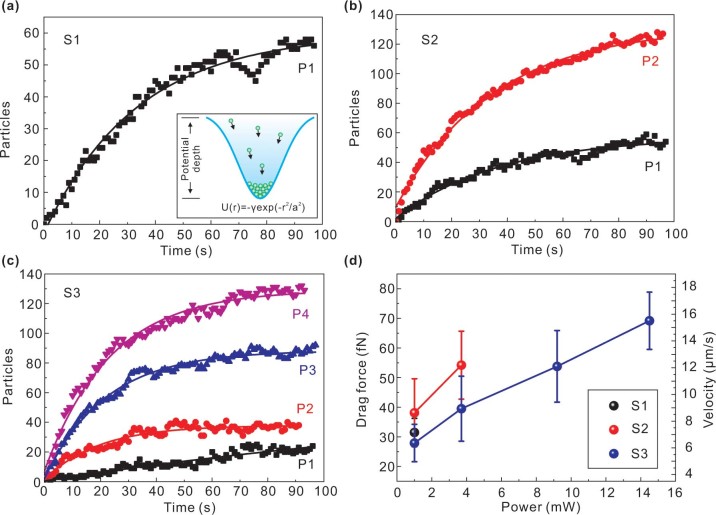
(a)–(c) Statistical analysis of the trapped PS as a function of time and laser power for S1-S3 respectively. Solid lines are the exponential fitting to experiment data by using the formula of *y* = *y*_0_ − *A* exp (−*R*_0_*t*) with *y*_0_ and *A* are constants, *R*_0_ is time coefficient. The inset in (a) shows the schematic of the trapping potential well with a Gaussian profile, whose depth is described as *U*(*r*) = −γexp(−*r*^2^/*a*^2^) where γ is a constant, *r* is the coordinate, and *a* is the width of the potential well. (d) Convective drag forces exerted on PS during trapping for S1-S3 respectively. The error bars indicate the experimental standard deviations.

Within the active state of the trapping mode, the number of PS particles increases exponentially until a plateau stage is achieved [Figs. 4(a)–4(c)]. This is in good agreement with the Gaussian profile of the trapping potential well [inset of Fig. 4(a)]^2^,^4^,^8^. Consequently, when PS particles are being drawn towards the trapping zone, they start to fill up the potential well from the bottom until it is fully occupied. An increase in potential well depth also means that the number of PS trapped within the zone will increase as the well volume tries to accommodate more particles. In particular, there is a small dip in the plot of Fig. 4(a) at t = 75 s. This is caused by a sudden crash of particles of considerable scale or instantaneous instability during the dynamic trapping process. This observation has a random character and such fluctuations are not present in repeated experiments.

Figure 4(d) shows the convective drag forces, based on the measured velocity data. The PS arrival velocities were time-averaged measurements obtained by monitoring their motion and analyzing the images frame by frame. Here we point out that accurate quantification on the contributions of optical and thermal forces will require much more extensive investigation as thermal forces under thermal gradient is also a direct consequence of local heating induced by optical field distribution as modified by plasmonic resonance in the nanoislands. The observed particle motion is essentially driven by long-range interactions originated from thermal convection. On the other hand, near-field plasmonic gradient force by its nature is due to short-range interaction. Consequently, one can quite reliably argue that thermal convection is the main contributor towards the measured drag force. Bearing in mind that the direction of measured drag forces is lateral, and the vertical upward drag force has the same magnitude as the lateral force^19^, thus the downward force should be larger than such drag force (with the addition of scattering force) in order to stabilize the trap. The used laser power densities are 8.8, 32.7, 81.3, and 128.2 *μW/μm*^2^ for P1–P4 respectively. These power levels are comparable to those used in plasmonic nanopyramidal-dimer arrays (10–40 *μW/μm*^2^)^9^, and is smaller than that of the cases of optical square lattice (110 μ*W/μm*^2^)^8^ and bowtie nanoantenna arrays (400 *μW/μm*^2^)^7^. The random AuNIS reported herein therefore holds exceptional prospect to serve as a highly efficient plasmonic tweezers for manipulating and organizing the desired shape and structure of micro/nano-sized objects. Our conclusion of high-efficiency, as observed from our experimental data, is based on comparison with the reported power densities required to realize a similar trapping behavior in patterned nano-fabricated nanostructures^7^,^8^,^9^. Quantitatively, the optical trapping efficiency of single-particle trap is defined as the product of maximum force exerted on the particle and speed of light in the medium over by the optical power at the focal plane^7^. However, the measured drag force in our case only represents the thermal convective drag force, and due to this limitation it is not possible at present to isolate the contribution from optical trapping for comparison with the data reported in Ref. 7.

**Figure 5:**
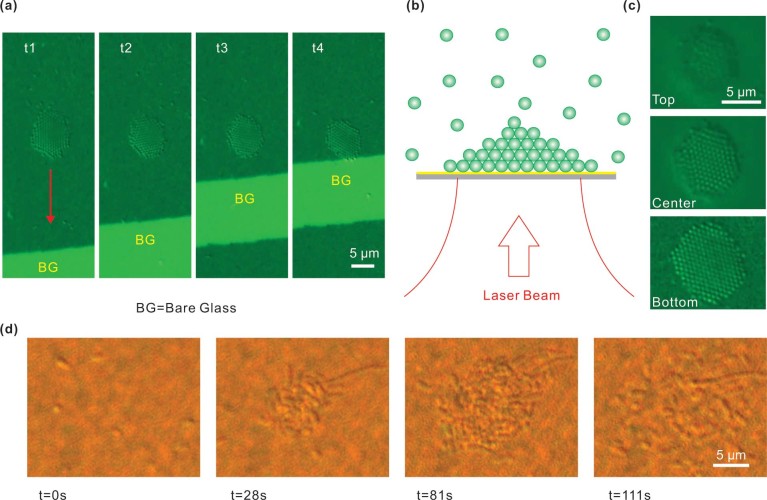
(a) Successive image frames showing the manipulation of trapped PS by moving the laser spot. The bright area denotes bare glass (BG) region. The red arrow denotes the direction of laser beam movement. Also see Supplementary Movie 2 as an example. (b) Schematic to show the stacking of trapped PS. (c) Image frames showing different layers of the stack. Also see Supplementary Movie 3 as an example. (d) Successive image frames showing the trapping of *Escherichia coli*. The laser beam was switched on at t = 0 s, and switched off before the last image. In all of the above cases, sample S2 and laser power level P2 (3.7 mW) were used.

In our experiment, the trapped PS can be readily moved to different areas of the AuNIS substrate as they follow the movement of laser spot [Fig. 5(a)]. Also, the trapped PS can even be manipulated desirably at any location within the boundary of the AuNIS (see Supplementary Movie 2 as an example). Such unique feature is not possible to be achieved using structured plasmonic system because of the fixed near-field pattern.

Within the trapping process, the PSs not only form a two-dimensional (2D) compact assembly, but they also stack vertically layer by layer into a three-dimensional (3D) tower cluster [Figs. 5(b) and 5(c) and see Supplementary Movie 3 as an example]. The stack has 3 to 5 layers, which corresponds to an effective trapping volume with a thickness around 2 μm. Also, we observe that only a few PSs are present in the top-most layer due to the weak trapping force at a distance from the AuNIS. The observed 2 μm thickness of trap is much larger than the range of near-field intensity (<100 nm^24^,^25^). On the one hand, this effect is attributed to a far-field intensity gradient force resulted from the structured scattering and refocusing of near-field radiation by the close-packed PS^7^,^26^. Furthermore, it is possible that other long-range trapping force exists instead of near-field optical gradient force.

PS particles with other sizes including 0.1, 1.0, and 1.5 μm (see Supplementary Fig. S2) have also been successfully trapped by the AuNIS. It is worth pointing out that the use of objective lenses with large magnification and high NA is not a necessary condition for trapping using AuNIS. High NA objectives are used merely for improved visualization of the trapped objects. Objectives with smaller magnification factor and lower NA can also be used to generate the trapping effect (see Supplementary Fig. S3). More importantly, we also realize trapping and assembling of living bacteria (*Escherichia coli*) [Fig. 5(d)], which has a typical size ranging from 1 to 8 μm, and effective refractive index is about 1.38. The bacteria assemble into a small cluster within 28 sec after switching the laser power from zero to P2 level, i.e. 32.7 *μW/μm*^2^. The trapped bacteria are arranged almost in only one layer dimension, i.e. without 3D stacking, due to the different force balance between optical and thermal forces as compared to the case of PS particles. The cells slowly return to random arrangement after the laser source is switched off. With this random AuNIS system, one can readily immobilize a target microorganism and move it to any specific local as desired without harming or damaging these biological cells. For example, one can use this system to select and guide the desired and specific sperm to interact with egg for various animal reproductive systems study.

## Discussion

**Figure 6:**
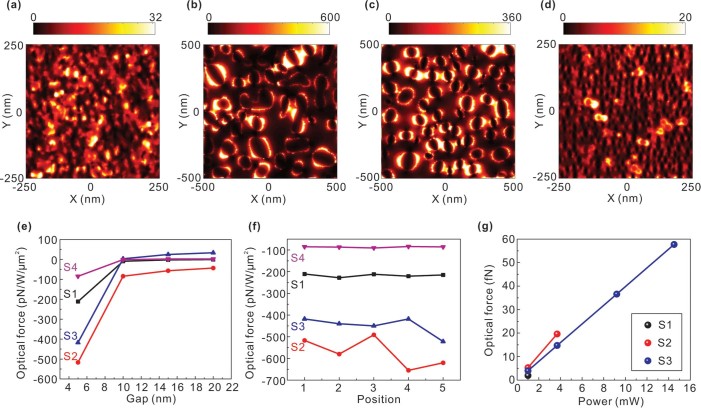
(a)–(d) Simulated electric field intensity distributions 5 nm above the upper surface of samples S1-S4, respectively. The light source is a plane wave with wavelength of 785 nm and X-polarized electric field. (e) Calculated optical forces exerted on a PS particle (diameter = 500 nm) with the gap between the PS and upper surface of AuNIS is varied from 5 to 20 nm. (f) Optical forces versus five random PS positions, with the gap between fixed at 5 nm. (g) Magnitude of optical trapping force versus the experimental laser power densities. For S1 and S2, only a part of results are shown for the purpose of comparing with the data shown in Fig. 4(d).

In order to further elucidate the trapping mechanism, and identify the respective contributions of optical and thermal forces to the observed particle trapping, we have performed numerical simulations for the near-field intensity distribution of AuNIS and force calculation exerted on the PS particles. The local electric field, as expected, is randomly distributed within the gap or around the boundary of nano-islands due to the excitation of surface plasmons and the interactive field coupling, with different field enhancements for samples S1–S4, respectively [see Figs. 6(a)–6(d) and Supplementary Fig. S4]. Particularly, for S1, the near-field is almost localized within the cracks and little optical field is scattered out of the surface. With the enhanced near-field, the optical trapping force exerted on the PS particle is greatly strengthened over the surface of nano-islands, but this force decays very fast away from the surface [Fig. 6(e)], which implies that the optical trapping force only gives rise to a short-range interaction between the nanostructure and the PS particles. The PS particle is randomly positioned above the surface with a fixed gap between, almost resulting in the same optical trapping force [Fig. 6(f)]. This is due to the collective effect of near-field from the LSP leading to the overall optical force. For S1-S3, the optical trapping forces are approximately −220 *pN/W/μm*^2^, −*600* *pN/W/μm*^2^ and −450 *pN/W/μm*^2^, respectively. The negative sign indicates that the force direction is towards the sample surface. Consequently, the optical trapping forces can be calculated according to the experimental laser power densities, and their magnitudes are shown in Fig. 6(g). We only present a part of results for comparison purposes. It can be seen that in all cases the overall optical trapping force is smaller than the convective drag force, as shown in Fig. 4(d). This means that the near-field optical force is unable to overcome the axial convective force, and the observed trapping phenomenon must involve contribution from another force. We attribute it to thermophoretic force which drags the PS particles from cold to hot zones, thus creating a net force that overcomes the axial drag force.

**Figure 7:**
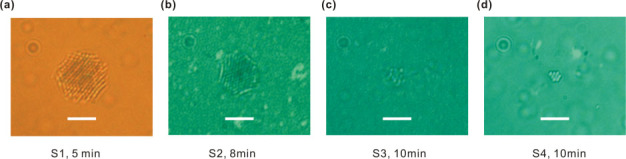
(a)–(d) Frame images of trapped PS assembly on samples S1-S4, respectively. The laser spot has been tightly focused on the sample surface with diameter as small as 1 μm. White bar: 5 μm.

To make the thermal effect more dominant for trapping, the laser spot on the sample surface is adjusted from 12 to 1 μm (see Supplementary Fig. S5). The frame images of trapped PS assembly on samples S1-S4 are shown in Figs. 7(a)–7(d), respectively. We can see that the particles are trapped over an area much larger than the 1 μm spot, especially for S1 and S2, whose area has a diameter of about 8 μm. This result suggests that those particles trapped outside the laser spot diameter are held in place by thermal forces other than optical trapping force. In this case, we can also realize trapping on S4 even though it is an off-resonance sample [Fig. 7(d)] together with the demonstration of trapping single, double, and three PS by adjusting the laser power (see Supplementary Movie 4). On the other hand, it is found that all the trapping phenomena vanish after adding a 1 μL droplet of salt solution (10 mM in concentration) into the PS solution (see Supplementary Movie 5). This result is supported by the fact that thermophoretic force is sensitive to local environment.

Overall, we attribute the trapping mechanism to the following processes (also see schematic illustration in Supplementary Fig. S6): (1) lateral motion that draws PS to the hot region due to convection; (2) vertical motion that pushes PS out of the region because of the axial convection; (3) near-field optical force due to plasmonic localization and thermophoresis that pushes the PS from cold to hot regions. Thus, the net force from process (3) must be larger than that from process (2) in order to stabilize and maintain the trapping zone.

We have estimated the magnitude of the optical trapping force through simulation, and the convective drag force experimentally. Thermophoresis is associated with the Soret coefficient and temperature gradient. The Soret coefficient can be obtained by measuring the thermo diffusion coefficient of particles in solution, and the temperature gradient can be obtained by measuring the three-dimensional temperature distribution around the nanostructures. All these measurements require careful design of the experiment. We are conducting ongoing experiments to determine the magnitude of thermophoretic forces. On the other hand, we propose to incorporate a heat sink layer^27^ directly underneath the nano-islands for efficient heat removal in order to ensure minimal thermal effects so that the optical force from the AuNIS may become dominant.

In brief, our large-scale AuNIS (22×22 mm^2^ in area), despite with randomness, can perform arbitrary trapping and moving of micro/nano-objects with ease just by focusing the laser beam at any location of the surface. It needs to be emphasized that our trapping scheme is almost geometry free and highly compatible to optical microscopes as opposed to the Kretschmann configuration^13^ in which the illumination has to be under total-internal reflection mode and the angle is limited to a narrow range. Despite that the reported thermal annealing scheme may not offer high degree of reproducibility in terms of feature sizes, the current trapping-by-AuNIS approach does not require precise control on feature parameters while the fabrication steps involved are extremely simple, unlike those used in the fabrication of well-defined nano-structures. Such unique advantages place our system to be more practical for nanotechnology and life science applications in terms of cost-effective fabrication, uniformity of localized near-field in microcosmic regime, ease of trapping and manipulating objects with micro and nanosizes. More importantly, our random AuNIS exhibits comparable trapping performance to the well-patterned systems. In addition, our random plasmonic system offers higher degree of freedom for the trapping tasks. For instance, if the low laser power density is of a concern for an experiment, one can use sample S1 and power P1 [8.8 *μW/μm*^2^, Fig. 4(a)] to resolve this problem. On the other hand, if more objects are needed to be trapped for specific research or application, one can use higher power such as P4 at S3 [130 PSs being trapped, Fig. 4(c)] for generating larger potential of trapping force in capturing and directing the desired objects to testing location.

In summary, we have proposed and demonstrated the feasibility of using large-scale random AuNIS for trapping and manipulating of PS particles and live *Escherichia coli* cells with ease. The employed laser power density can be as low as several *μW/μm*^2^. The near-field optical trapping force is superimposed with a long-range thermophoretic force which pushes the PS particles from cold to hot regions. Therefore the resulting force exceeds that due to axial convective drag, and the net effect results in a trapping force pointing towards the surface. Such example shows that the system can be extended for virology and immunology testing of infected biological fluid samples. We anticipate that the reported random plasmonic system here can be integrated into advanced optofluidic devices for applications such as on-chip manipulation of nano- or micro-sized objects, cancer cells sorting and light-controlled reactions between targeted microorganisms.

## Methods

### Fabrication of AuNIS

The fabrication of AuNIS by thermal evaporation and anneal processing has been widely studied^17^,^28^. The morphology of the AuNIS can be easily modified by varying the nominal thickness and processing conditions (anneal temperature and time), leading to a broad tunable resonant band (520–900 nm)^28^. In our case, the gold thin film was deposited on micro cover glasses (Ted Pella Inc.) by a thermal evaporation system (Edwards, UK). The base pressure was 10^−6^ Torr. The deposition rate was kept at 0.1 Å/s, as monitored by a quartz crystal microbalance (Maxtek Inc.). Four different AuNIS samples S1–S4 were fabricated, with nominal thickness of 8, 10, 10, and 8 nm, respectively. S1 was used as-deposited with no thermal anneal. The thermal anneal conditions for S2, S3, and S4 were 400^o^C for 12 minutes, 550^o^C for 3 hours, and 550^o^C for 12 hours, respectively. The as-prepared samples were stored in atmospheric environment.

### Optical setup

Our optical setup is schematically shown in Fig. 1(c), which is based on the use of a Nikon inverted microscope (TE2000-U). A linearly polarized laser beam (λ = 785 nm) from a semiconductor diode is guided to the AuNIS surface via an oil-immersion objective [60×, numerical aperture (NA) = 1.49]. The image is captured with a CCD camera through the same objective lens by using the differential interference contrast technique. The laser spot on the sample surface as measured from the CCD image has a diameter of 12 μm. The laser source is from a micro-Raman module attached to the microscope. The beam is not expanded to fill the objective lens aperture. Hence the laser spot is not focused to the ultimate spot size achievable by the objective. The laser power at the sample surface is adjustable between five levels, with P1–P5: 1.0, 3.7, 9.2, 14.5, and 41.6 mW. A band-reject filter is inserted to block the trapping laser beam. The targets are polystyrene spheres (PS) with a diameter of 0.5 μm (Polysciences Inc.). The PSs are suspended in deionized water and the concentration is about 1.1×10^9^ particles/mL. During a typical trapping experiment, a 20 μL droplet of the suspension is placed directly on the AuNIS surface.

### Temperature measurement

The temperature measurement was based on measuring the fluorescence efficiency of Rhodamine B (0.1 mM solution) whose temperature dependence has been well documented^29^. In the measurements, a 532 nm green laser was used to excite the fluorescence. A spectrometer (Ocean Optics 2000) was used to collect the fluorescent spectrum. The calibration data and their fitting plot are provided in Supplementary Fig. S7. The fitting is based on the formula ln[I(*T*)/I(*T_ref_*)] = *β*(1/*T*−1/*T_ref_*), where I(*T*) is the integral fluorescent intensity at temperature *T*, *T_ref_* = 294*K* (21^o^C) is the room temperature, and is a proportional constant, respectively. Such calibration was repeated three times to get an averaged *β* = 2372*K*, which is used to determine the temperatures on all the samples when illuminated by different 785 nm laser powers. For each AuNIS sample and a certain laser power, the temperature measurement was repeated five times, and the standard deviations of such five measurements are shown as error bars in Fig. 3(a).

### Drag force calculation

The arrival velocities of PS were measured by monitoring their motion and analyzing the images frame by frame. The drag forces exerted on the PS are calculated by applying the Stokes law *F = μν*, where *μ* = 3*πηD* is the drag coefficient with *η* the dynamic viscosity and *D* the diameter of the PS, and ν is the velocity of particle^1^,^7^. The temperature dependence of *η* is also considered in the calculation^30^.

### Theoretical optical force calculation

Three-dimensional finite-difference time-domain (FDTD) simulations were performed to obtain the near-field intensity distribution in the vicinity of samples S1–S4. The 2D patterns of S1-S4 from SEM images [Fig. 1(a)] were loaded into the modeling, and the heights of particles were defined according to the atomic force microscope images (see Supplementary Fig. S8). The averaged heights of particles for S1–S4 are about 5, 30, 40, and 15 nm, respectively. The PS particle having diameter of 500 nm was placed on the top of AuNIS with the gap between ranging from 5 to 20 nm. After obtained the electromagnetic field distributions in the simulation region, the Maxwell stress tensor method was utilized to calculate the optical force exerted on the PS particle. Note that the calculated optical force is the sum of optical gradient force and scattering force.

## Supplementary Material

Supplementary Information Supplementary Information

Supplementary Information Supplementary Movie 1

Supplementary Information Supplementary Movie 2

Supplementary Information Supplementary Movie 3

Supplementary Information Supplementary Movie 4

Supplementary Information Supplementary Movie 5
