# Supplementary material for: Trapping and assembling of particles and live cells on large-scale random gold nano-island substrates
Source: Sci Rep. 2015 Apr 30;5:9978. doi: 10.1038/srep09978 (PMC5386207; doi:10.1038/srep09978)
Supplement: Supplementary Information [file srep09978-s1.pdf]

Supplementary Information

Trapping and assembling of particles and live cells on large-scale random gold nano-island substrates

Zhiwen Kang[1], Jiajie Chen[1], Shu-Yuen Wu[1], Kun Chen[1], Siu-Kai Kong[2], Ken-Tye Yong[3], Ho-Pui Ho[1,a)]

[1]Centre for Advanced Research in Photonics, Department of Electronic Engineering, The Chinese University of Hong Kong, Shatin, NT, Hong Kong SAR, China

[2]Program of Biochemistry, School of Life Sciences, The Chinese University of Hong Kong, Shatin, NT, Hong Kong SAR, China

[3]School of Electrical and Electronic Engineering, Nanyang Technological University, Singapore 639798, Singapore

[a)]Corresponding e-mail address: hpho@ee.cuhk.edu.hk

1. TABLE:

Table S1. Trapping states of S1-S4 at different laser powers P1-P5

Sample\power	P1 (1.0mW)	P2 (3.7mW)	P3 (9.2mW)	P4 (14.5mW)	P5 (41.6mW)
**S1**	**Trap**	Heat	Heat	Bubble	Bubble
**S2**	**Trap**	**Trap**	Heat	Heat	Bubble
**S3**	**Trap**	**Trap**	**Trap**	**Trap**	Heat
**S4**	no	no	no	Scatting	Scatting

“Heat” refers to thermal convection is too strong to maintain the trap.

“Scattering” refers to PS particles are pushed out of focus due to scattering force.

2. FIGURES:

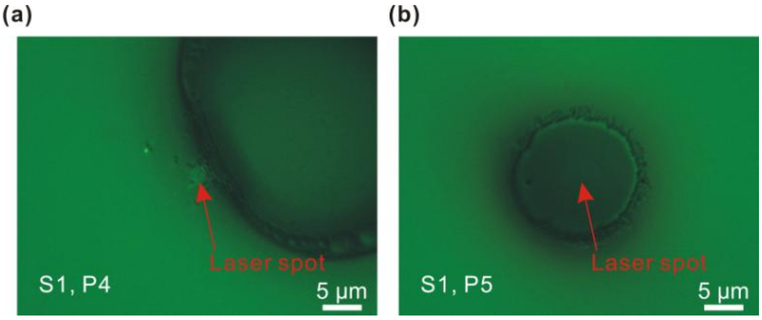

Fig. S1. Bubble generation in S1 when the laser power is at P4: 14.5 mW (a) and P5: 41.6 mW (b), respectively.

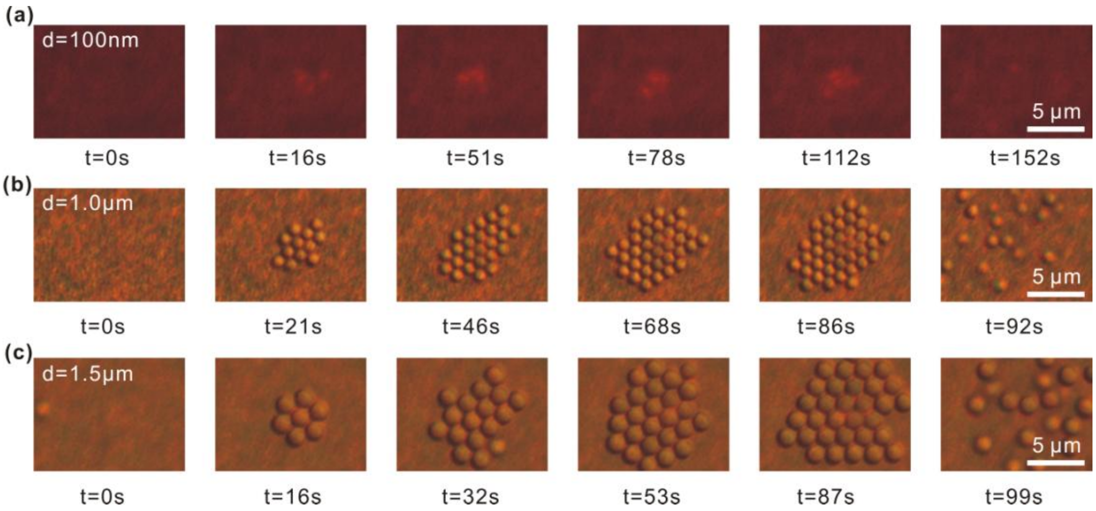

Fig. S2. (a)-(c) Successive images showing the trapping processes of PS with different diameters of 0.1, 1.0, and 1.5 μm, respectively. For case in (a), the 0.1 μm fluorescent PS (Invitrogen Inc.) is excited by a green laser (λ=532 nm) for the purpose of observation. This green laser beam is filtered before entering the CCD. Such 0.1 μm PS seems to be loosely trapped due to the weak trapping force exerted on it (optical gradient force scales with the third power of the particle size). In all cases, the AuNIS sample S2 and power P2 (3.7 mW) are used. The laser beam was turned on at t=0 s and turned off before the last image. The concentrations of 0.1, 1.0, and 1.5 um PSs are 3×10^{9}, 1.4×10^{8}, and 4×10^{7} particles/mL,

respectively.

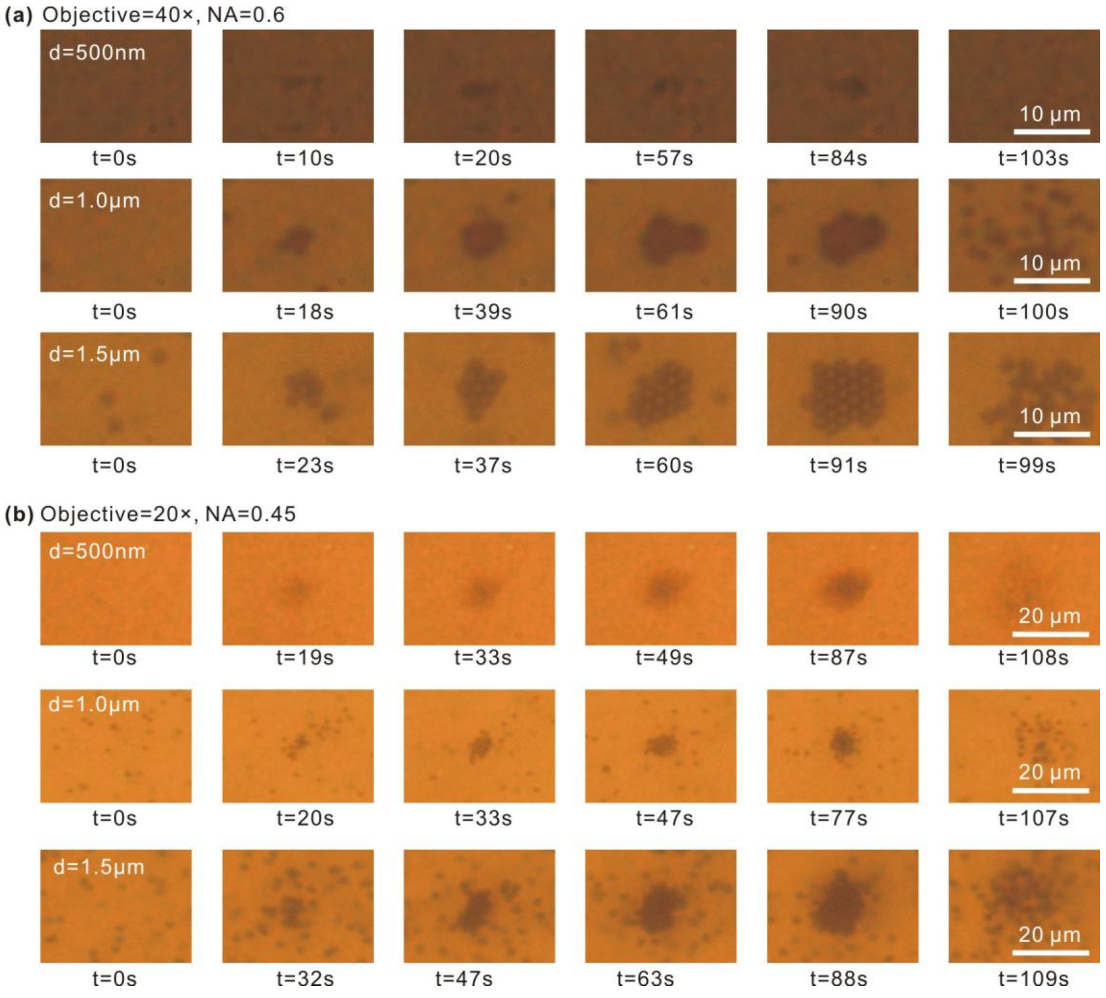

Fig. S3. Successive images of the trapping processes as different objectives are used. (a) Objective=40×/NA=0.6. (b) Objective=20×/NA=0.45. The PS with different diameters (0.5, 1.0, 1.5 μm) were trapped. Because the magnification is relatively small, the PS was seen like a blur. However, one can still observe the accumulation of the PS during trapping. In all cases, the AuNIS sample S2 and power P2 (3.7 mW) were used. The laser beam was turned on at t=0 s and turned off before the last images.

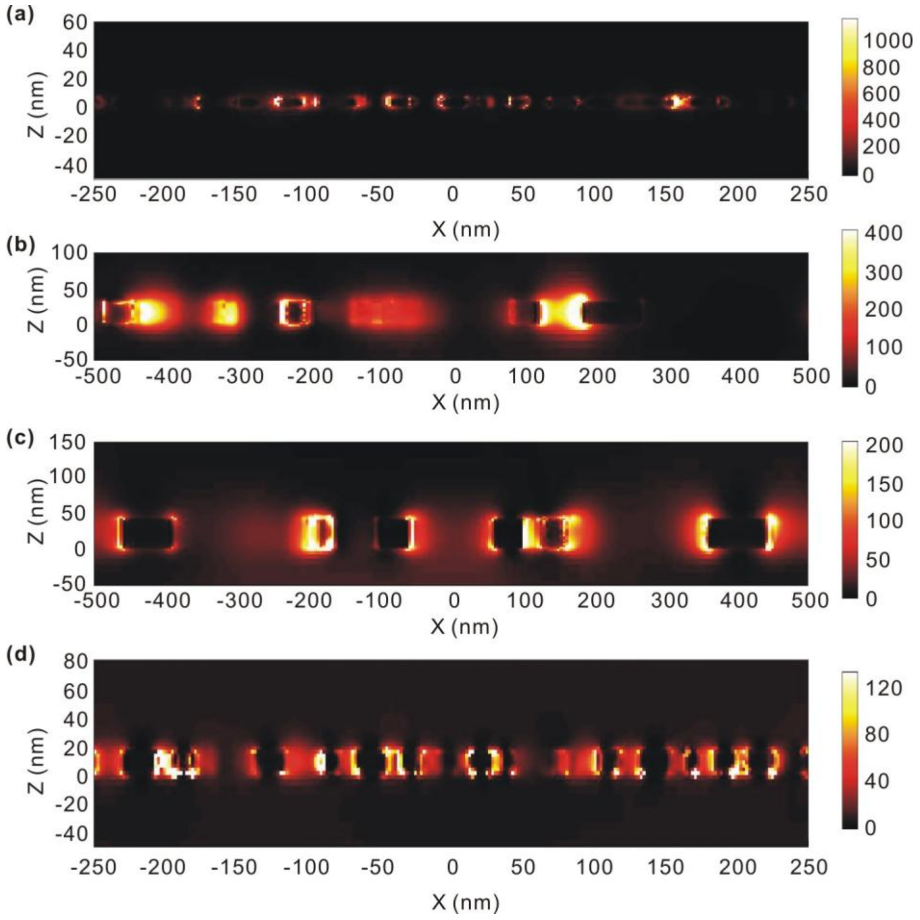

Fig. S4. (a)-(d) Simulated electric field intensity distributions on the XZ plane (Y=0) for samples S1-S4, respectively. The incident wavelength is 785 nm (plane wave) and electric field is X-polarized.

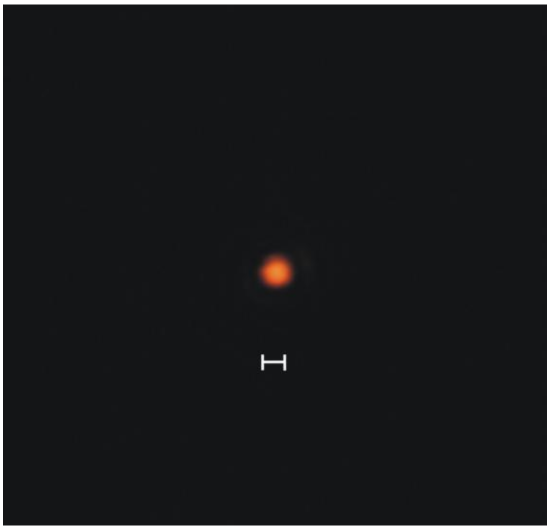

Fig. S5. Laser spot shining on the sample surface imaged by CCD camera. Scale bar: 1 μm.

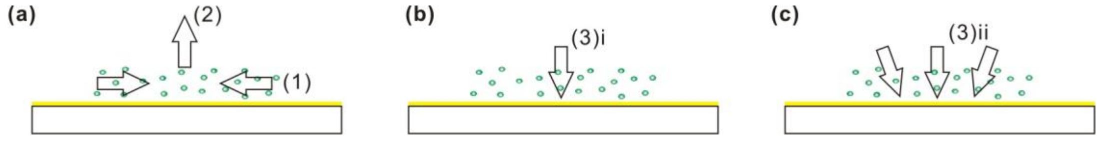

Fig. S6. (a) Thermal convection including lateral motion (1) and vertical motion (2). (b) Near-field optical trapping force (3)i. (c) Thermophoretic force pushing the PS particles from cold to hot. The calculated optical trapping force itself alone is less than the axial convective drag force $\left|F_{(3)\mathrm{i}}\right| < \left|F_{(2)}\right|$. Thermophoresis assists the optical trapping and the resulting force is larger than the axial convective drag force $\left|F_{(3)\mathrm{ii}}\right| + \left|F_{(3)\mathrm{i}}\right| > \left|F_{(2)}\right|$.

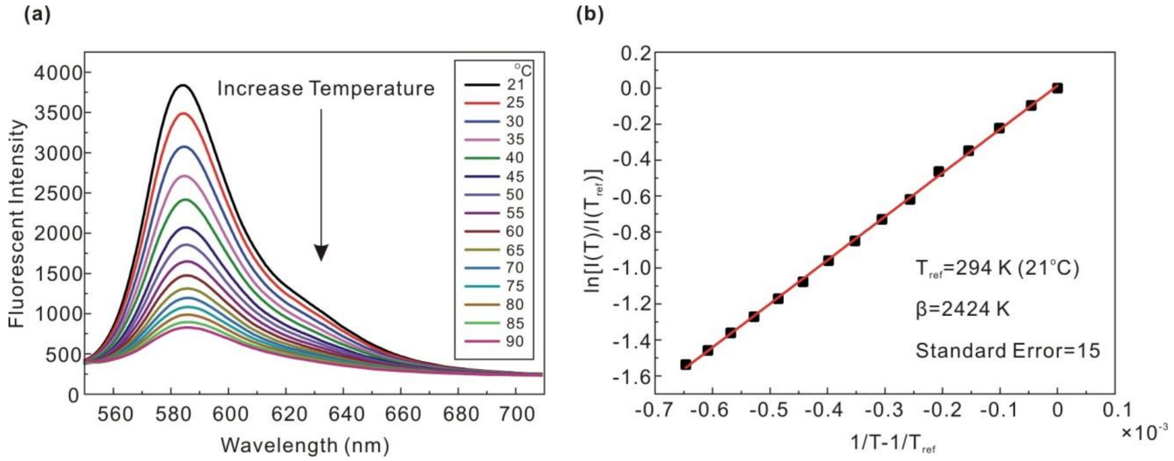

Fig. S7. (a) Fluorescent spectra of Rhodamine B solution (concentration: 0.1 mM) at different temperatures. (b) Fitting of the experiment data.

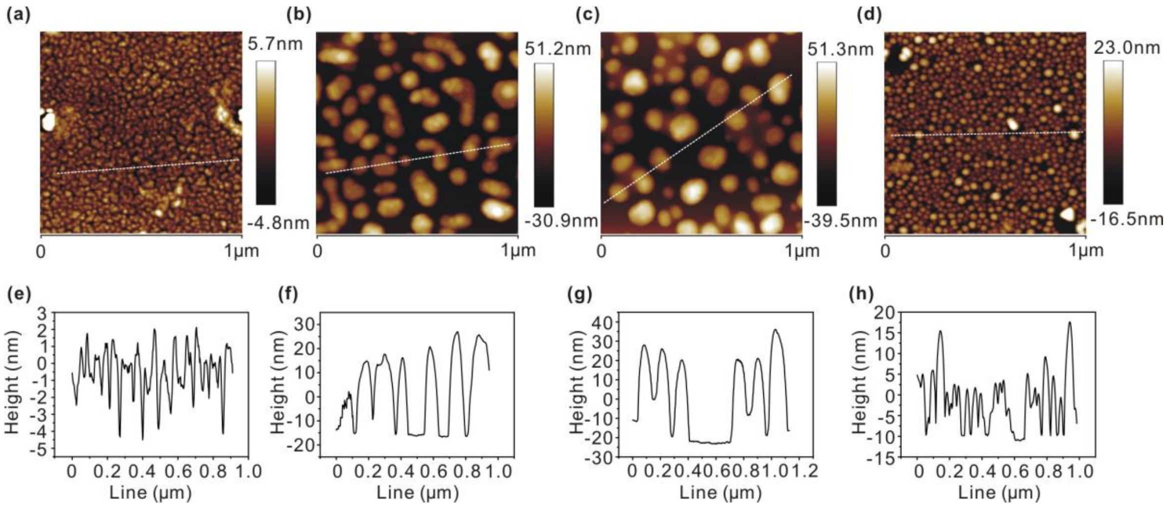

Fig. S8. (a)-(d) Atomic force microscope images, and (e)-(h) the height profiles along the white dashed line in the AFM images for samples S1-S4, respectively.

3. <u>MOVIES</u>

Movie 1: Trapping of 0.5 μm PS on S2 at P2. Laser spot diameter is 12 μm.

Movie 2: Manipulation of trapped PS on S2 at P2. Laser spot diameter is 12 μm.

Movie 3: Changing the image plane to see different layers of stacking. Substrate is S2 and incident power is P2. Laser spot diameter is 12 μm.

Movie 4: Trapping of single, double, and three PS on S4. The laser spot diameter is 1 μm.

Movie 5: Vanishing of trapping phenomena for S1-S4, by adding a 1 μL droplet of NaCl solution (concentration: 10 mM). Laser spot diameter is 1 μm.
